# Reduced bilateral auditory cortex activation during pure-tone perception in pediatric HIV

**DOI:** 10.3389/fnins.2026.1623144

**Published:** 2026-04-21

**Authors:** Keri J. Woods, Tzy-Jyun Yao, Barbara Laughton, Ernesta M. Meintjes, Peter Torre, Marcin Jankiewicz

**Affiliations:** 1 Biomedical Engineering Research Centre (BMERC), Division of Biomedical Engineering, Department of Human Biology, University of Cape Town, Cape Town, South Africa; 2 Neuroscience Institute, University of Cape Town, Cape Town, South Africa; 3 Centre for Biostatistics in AIDS Research, Harvard T.H. Chan School of Public Health, Boston, MA, United States; 4 Family Centre for Research with Ubuntu, Department of Pediatrics and Child Health, Stellenbosch University, Cape Town, South Africa; 5 Cape Universities Body Imaging Centre (CUBIC), Faculty of Health Sciences, University of Cape Town, Cape Town, South Africa; 6 School of Speech, Language, and Hearing Sciences, San Diego State University, San Diego, CA, United States

**Keywords:** ART, auditory cortex, auditory processing, central auditory system, children with perinatal HIV (CPHIV), functional MRI (fMRI), pediatric HIV, pure tones

## Abstract

**Introduction:**

Children with perinatally acquired HIV (CPHIV) are at increased risk of neurodevelopmental difficulties, including hearing-related impairments, despite early initiation of antiretroviral therapy (ART). Previous studies have reported a higher prevalence of hearing loss in CPHIV compared with uninfected children; however, the contribution of the central auditory system to these auditory differences remains unclear. Understanding central auditory processing in CPHIV is important, as even subtle auditory difficulties during childhood can negatively affect speech and language development, academic performance, and quality of life.

**Methods:**

Functional MRI was used to examine neural responses to auditory stimulation in 108 11-year-old children (60 CPHIV and 48 children without HIV). During scanning, participants listened to pure tones at low (500 Hz), middle (1,500 Hz), and high (4,000 Hz) frequencies.

**Results:**

CPHIV demonstrated modestly elevated hearing thresholds (reflecting poorer hearing sensitivity) at several frequencies; however, the prevalence of clinically defined hearing loss did not differ between groups. Across all children, pure-tone stimulation elicited robust bilateral activation of the auditory cortices, with both the spatial extent and magnitude of activation decreasing as tone frequency increased. Relative to controls, CPHIV exhibited significantly reduced bilateral auditory cortex responses across frequencies. These group differences persisted after accounting for sex and handedness and after excluding children with hearing loss. Associations between hearing thresholds and auditory cortex activation were generally weak, except at 4,000 Hz in CPHIV, where poorer hearing was associated with stronger auditory cortex activation, consistent with a compensatory neural response.

**Discussion:**

Despite largely normal peripheral hearing, CPHIV receiving ART exhibited reduced bilateral auditory cortex responses during pure-tone processing. These findings suggest that alterations within the central auditory system may contribute to auditory vulnerability in CPHIV.

## 1 Introduction

With the introduction of combined antiretroviral therapy (cART), HIV infection has shifted from a fatal illness to a chronic condition. However, cART does not eradicate the virus (Le Douce et al., 2012). HIV is known to cross the blood–brain barrier (BBB) soon after infection, and because antiretroviral drugs only partially penetrate the BBB (Ene et al., 2011), the central nervous system can serve as a viral reservoir (Schrager and D'Souza, 1998; Pomerantz, 2001).

Owing to the rapid and extensive brain development early in life, children with perinatally acquired HIV (CPHIV) are at greater risk of neurological impairment compared with individuals who acquire HIV later in life. In children, HIV infection has been associated with a range of neurocognitive difficulties, including reduced processing speed (Koekkoek et al., 2008; Ruel et al., 2012; Smith et al., 2012), memory deficits (Smith et al., 2012), and impaired motor skills (Knight et al., 2000; Blanchette et al., 2002). Additional challenges include attentional difficulties (Ruel et al., 2012), developmental delays (van Arnhem et al., 2013), and notably, impairments in verbal and language abilities (Brackis-Cott et al., 2009; Smith et al., 2012; van Arnhem et al., 2013; Redmond et al., 2016).

Hearing-related difficulties represent a relatively underrecognized complication of HIV infection (Matas et al., 2006; Torre III et al., 2012). Reported prevalence estimates of hearing loss in CPHIV vary widely, ranging from 6% to 84% (Dawood et al., 2020), and the mechanisms underlying these auditory difficulties remain poorly understood. Understanding hearing-related difficulties in children is particularly important, as disruptions in auditory processing can adversely affect speech and language development (Davis et al., 1986), with broader implications for communication, social functioning, and academic performance.

The auditory pathway comprises peripheral and central components, including the outer ear, middle ear, inner ear, and the central auditory system. The central auditory system encompasses the cochlear nuclei and superior olivary complex in the brainstem, the inferior colliculus in the midbrain, the medial geniculate nucleus of the thalamus, and the auditory cortex located in the superior temporal gyrus (Martin et al., 2004).

Auditory processing is organized tonotopically, such that different regions of the system respond preferentially to different sound frequencies. This tonotopic organization originates in the cochlea and is preserved throughout the central auditory pathway (Bilecen et al., 1998). The primary auditory cortex (PAC), which forms the core of the auditory cortex, is situated in Heschl's gyri within the superior temporal gyrus (Martin et al., 2004; Pickles, 2013). Functional MRI (fMRI) studies have consistently demonstrated robust involvement of the PAC in auditory processing across a range of stimuli, including pure tones and speech (Dhankhar et al., 1997; Grady et al., 1997; Bilecen et al., 1998; Jäncke et al., 1998; Binder et al., 2000). Although auditory stimulation typically elicits bilateral activation of the auditory cortices (Binder et al., 1994, 2000), some studies have reported left-lateralized responses (Millen et al., 1995; Bilecen et al., 1998). The tonotopic organization of the PAC has been repeatedly demonstrated using fMRI (Bilecen et al., 1998; Le et al., 2001; Formisano et al., 2003; Talavage et al., 2004; Yetkin et al., 2004; Langers et al., 2007).

CPHIV have been reported to show a higher prevalence of hearing loss compared with children without HIV (Matas et al., 2006; Torre III et al., 2012), suggesting potential disruption along the auditory pathway. CPHIV are also more susceptible to recurrent middle ear infections (Principi et al., 1991; Shapiro and Novelli, 1998; Taipale et al., 2011), which can result in conductive hearing loss (Chao et al., 2012). Several studies have found that hearing loss in CPHIV is predominantly conductive, consistent with recurrent middle ear pathology (Taipale et al., 2011; Chao et al., 2012; Hrapcak et al., 2016). In contrast, other investigations have reported a higher prevalence of sensorineural hearing loss—reflecting damage to the cochlea or the cochlear portion of the vestibulocochlear nerve (Haines, 2004)—in this population (Torre III et al., 2012; Christopher et al., 2013). Collectively, these findings suggest that hearing loss in CPHIV may arise from multiple levels of the auditory pathway, extending beyond middle ear disease.

Cochlear function in CPHIV has been examined using distortion product otoacoustic emissions (DPOAEs), which reflect outer hair cell integrity (Abdala and Visser-Dumont, 2001). While several studies have reported no association between HIV status and DPOAE measures (Torre III et al., 2015a,b), others have observed reduced DPOAE levels in CPHIV relative to uninfected controls (Maro et al., 2016). Together, these findings point to mixed evidence regarding cochlear involvement.

In addition to peripheral mechanisms, deficits may also arise within the central auditory system (CAS). Although Matas et al. (2008) did not directly assess central auditory processing, their behavioral audiometry findings are consistent with the possibility that CAS dysfunction contributes to hearing-related difficulties in young CPHIV. While auditory brainstem responses (ABR) have been used to investigate HIV-related abnormalities of the auditory nerve and brainstem auditory pathway (Matas et al., 2006; Torre III et al., 2019), relatively little research has examined the impact of HIV on more central components of the auditory system in children.

HIV is known to affect brain structure and function during development. Neuroimaging studies of children and adolescents with HIV have reported white matter atrophy (Sarma et al., 2014; Cohen et al., 2016; Nwosu et al., 2018), alterations in regional gray matter volume (Sarma et al., 2014; Cohen et al., 2016; Nwosu et al., 2018), and changes in subcortical volumes (Randall et al., 2017; Nwosu et al., 2018). Additional findings include ventricular enlargement (van Arnhem et al., 2013), white matter lesions (van Arnhem et al., 2013), altered cortical thickness and gyrification (Yadav et al., 2017; Nwosu et al., 2018, 2021), and abnormalities in white matter microstructure (Uban et al., 2015; Jankiewicz et al., 2017; Hoare et al., 2018). Other reported abnormalities include atypical resting-state connectivity (Toich et al., 2018), altered metabolite concentrations (Robertson et al., 2018), calcifications (Govender et al., 2011), and subcortical shape deformation (Lewis-de los Angeles et al., 2016). Notably, several of these alterations involve temporal lobe regions near the auditory cortex. For example, CPHIV have shown reduced gyrification in right temporal regions (Nwosu et al., 2018) and reduced cortical thickness in the right superior temporal region (Yadav et al., 2017). Furthermore, a resting-state fMRI study reported increased connectivity between a seed in the right postcentral gyrus and left temporal regions, including the superior temporal lobe, in CPHIV relative to control children (Toich et al., 2018).

Given the widespread effects of HIV on brain development, it is plausible that the hearing-related difficulties observed in CPHIV partly reflect differences in neural activity within the auditory cortices.

In the present study, we used fMRI to examine the blood–oxygen-level-dependent (BOLD) responses across the brain, with a particular focus on the auditory cortices, while CPHIV and children without HIV listened to pure tones. To our knowledge, this is the first pediatric fMRI study to directly compare neural responses to auditory stimulation between these groups. Based on prior reports of increased hearing-related vulnerability in CPHIV (Torre III et al., 2012), we hypothesized reduced task-related auditory cortex activation in CPHIV relative to children without HIV.

## 2 Materials and methods

### 2.1 Participants

We acquired data from 137 children as part of the Auditory Research in Children with HIV: Cape Town (ARCH: Cape Town) study, conducted between 2016 and 2019 to examine the effects of HIV and ART on various aspects of the auditory system, from the periphery to the auditory cortex. Of these, 78 were CPHIV recruited from the Children with HIV Early Antiretroviral Therapy (CHER) trial (Violari et al., 2008; Cotton et al., 2013), and 59 age-matched control children without HIV were recruited from similar neighborhoods in Cape Town, South Africa. Inclusion criteria for the CHER trial (from which CPHIV were accrued) included age 6–12 weeks, HIV infection, and CD4% > 25%. Exclusion criteria for the CHER trial and controls included birth weight < 2,000 g and CNS problems (not due to HIV) or dysmorphic syndromes.

The CPHIV from the CHER trial have been followed since enrollment at under 12 weeks old. The infants were randomized to one of three treatment groups: early treatment, with immediate initiation of ART and planned interruption after either 40 or 96 weeks (40 W or 96 W arms, respectively), with treatment restarting when clinical and/or immunological criteria were met; or late treatment (after 12 weeks), starting ART only when clinically indicated or when CD4% dropped below 20% (or 25% in the first year). One child with CD4% < 25% was randomized into one of the early treatment groups (Violari et al., 2008; Cotton et al., 2013). In this paper, all these children were combined into one CPHIV group. The control children without HIV included both children who were HIV-unexposed and uninfected, born to HIV-seronegative mothers (CHUU), and exposed and uninfected children born to mothers living with HIV (CHEU). Control children were recruited from a linked vaccine trial and came from similar communities as the CPHIV.

This study was approved by the Human Research Ethics Committees of the participating institutions. Parents/guardians provided written informed consent, and children provided oral assent.

### 2.2 Hearing data

The audiologist conducting the hearing assessments was blinded to the children's HIV status. Pure-tone air-conduction thresholds were measured separately in each ear at frequencies ranging from 250 to 8,000 Hz. The intensity of each tone was increased in 5 dB increments until the child could reliably detect it. The hearing threshold was defined as the lowest intensity (in dB) at which the child could hear the tone, with higher thresholds indicating poorer hearing.

Hearing loss in an ear was defined as a pure-tone average (PTA) across 500, 1,000, 2,000, and 4,000 Hz exceeding 15 dB. Each child was assigned a binary score (1 = presence, 0 = absence) for four categories: hearing loss in both ears, hearing loss in the left ear only, hearing loss in the right ear only, and no hearing loss.

Shapiro-Wilk tests indicated that the hearing threshold data were not normally distributed (all *p* < 0.0001). Between-group comparisons of thresholds were therefore conducted using Mann-Whitney U (Wilcoxon rank-sum) tests. Differences in the categorical hearing loss variables between control and CPHIV groups were assessed using Fisher's exact tests.

### 2.3 Imaging protocol

All scans were acquired on the 3T Skyra MRI (Siemens Medical Systems, Erlangen, Germany) at the Cape Universities Body Imaging Center (CUBIC) in Cape Town, South Africa, with a 32-channel head coil.

Anatomical images were acquired in the sagittal orientation using a multi-echo magnetization prepared rapid gradient echo (MEMPRAGE) sequence (van der Kouwe et al., 2008) (176 slices, repetition time (TR) 2,530 ms, TEs 1.69/3.54/5.39/7.24 ms, TI 1,100 ms, slice thickness 1 mm, 224 x 224 x 176 mm^3^ field-of-view, resolution 1.0 × 1.0 × 1.0 mm^3^, duration 5 min 21 s).

The fMRI protocol consisted of two runs of 69 volumes each, acquired using sparse sampling (Liem et al., 2012; Perrachione and Ghosh, 2013) with a T2^*^-weighted gradient echo, echo planar imaging sequence (EPI; TR 4,000 ms, acquisition time (TA) 2,000 ms, TE 30 ms, 33 interleaved slices, 4 mm slice thickness, 250 x 250 x 164 mm^3^ field of view, resolution 3.0 x 3.0 x 4.0 mm^3^, with a 1 mm slice gap, duration 4 min 50 s). The auditory stimuli were presented during the first 2 s of the TR, uninterrupted by scanner noise, after which the data were acquired in the next 2 s.

### 2.4 Details of the fMRI task

Following the acquisition of six initial fMRI volumes, the auditory task began. The task consisted of 63 trials, including 15 silent trials and 48 auditory tone trials. Each trial lasted for 2 s, with a 2 s interstimulus interval (ISI), during which EPI data were acquired. Tones were delivered at low (500 Hz), middle (1,500 Hz), or high (4,000 Hz) frequencies to either the left or right ear at an intensity of 120 dB sound pressure level (SPL).

**Figure 1:**
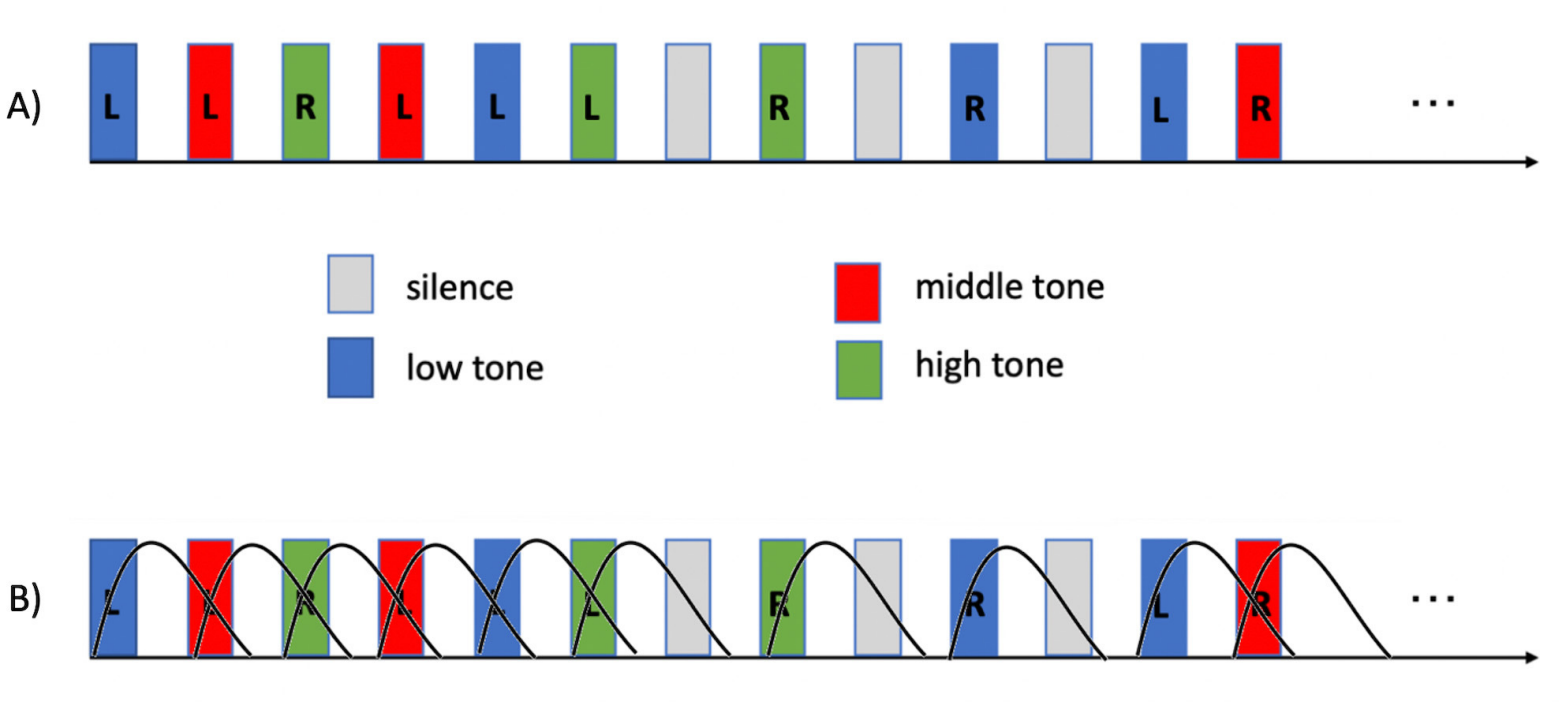
**(A)** Example segment of the auditory task. The task comprised 63 trials: 48 auditory tone trials and 15 silent trials. Auditory tones were presented for 2 s at low (500 Hz), middle (1,500 Hz), or high (4,000 Hz) frequencies to either the left (L) or right (R) ear. Each tone frequency was presented eight times per ear, along with 15 silent trials of equal duration, in randomized order. The task was administered twice to each child. **(B)** Example of the task model generated by convolving the boxcar representation of the stimulus timing with a canonical hemodynamic response function.

Children were instructed to indicate the ear in which they perceived each tone by pressing the corresponding button on a response pad. No response was required during silent trials. Each tone frequency was presented eight times to each ear, and tone and silent trials were pseudorandomized across the run. Importantly, no data acquisition occurred during tone presentation, allowing auditory stimuli to be perceived without interference from scanner noise. Each child completed two runs of the task. Task timing is illustrated in Figure 1A.

Behavioral responses were recorded for all trials, and accuracy across the 63 trials was calculated for each run. Accuracy values from the two runs were averaged to generate a single behavioral performance measure per child, which was then compared between diagnostic groups (CPHIV and control).

### 2.5 Preprocessing of fMRI data

**Figure 2:**
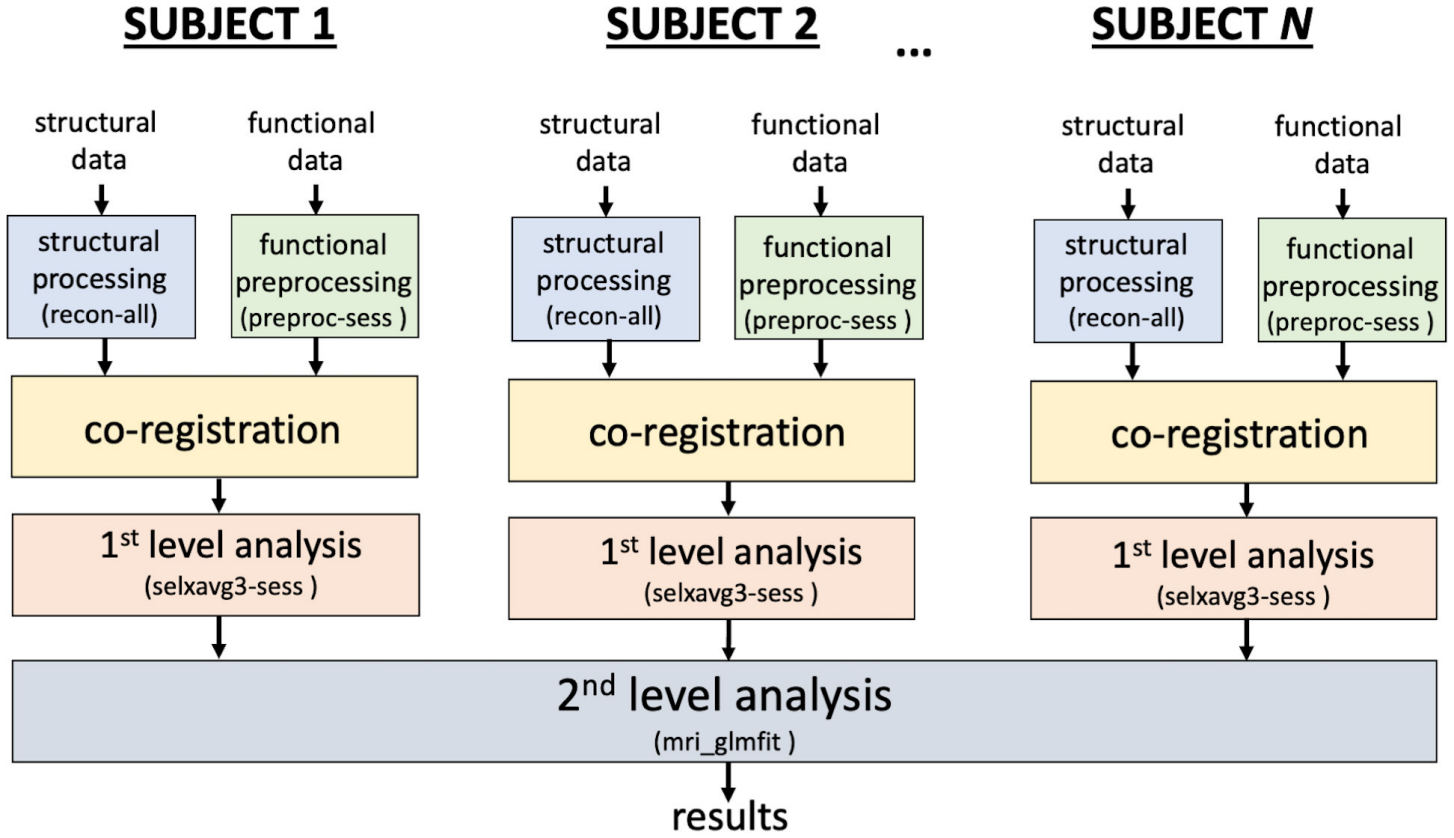
Overview of the fMRI analysis workflow implemented in FreeSurfer.

MRI data were preprocessed using FreeSurfer 6.0.1 (http://surfer.nmr.mgh.harvard.edu/). The workflow for processing the data is shown in Figure 2.

The anatomical scan for each participant was used to reconstruct cortical surfaces using the *recon-all* pipeline. FreeSurfer's Functional Analysis Stream (FS-FAST) (http://FreeSurfer.net/fswiki/FsFast) was used for all functional analyses. The six initial dummy functional volumes acquired before the task began were excluded from all analyses. The standard FS-FAST preprocessing pipeline was used for each participant, except that slice-time correction was not performed as the data were discontinuous due to the sparse sampling (Perrachione and Ghosh, 2013). Preprocessing included motion correction, creation of a registration template, functional-anatomical registration using boundary-based registration with six degrees of freedom (Greve and Fischl, 2009), mask creation, intensity normalization, resampling raw time series to MNI305 (Collins et al., 1994) space (an atlas based on an average of 305 anatomical MRI scans) and FreeSurfer's fsaverage surface left (LH) and right (RH) spaces, and spatial smoothing with a FWHM of 5 mm.

For each child, we analyzed data from the maximum number of consecutive measurements with no movement greater than 3 mm displacement or 3.0° rotation. A run was excluded from further analyses if the section of usable data did not include at least three events from each condition (i.e., low, middle, high, and silent). For three children, only one run was included in the analysis.

### 2.6 Head motion during scanning

Although FreeSurfer provides motion correction outputs, AFNI tools were used to derive volume-wise estimates of head motion. Raw functional data were processed with AFNI's *3dvolreg* to estimate six rigid-body motion parameters for each volume. AFNI's *1d_tool.py* was then used to compute the Euclidean norm of the motion parameters (Enorm) and the maximum pairwise displacement for each run.

Two summary motion metrics were calculated for each child: (1) average volume-wise head motion, defined as the mean Enorm across both runs, and (2) maximum pairwise displacement, defined as the larger of the two run-specific maximum displacement values. Shapiro-Wilk tests indicated that both motion measures were non-normally distributed (both *p* < 0.0001). Accordingly, group differences between CPHIV and control children were evaluated using Wilcoxon rank-sum tests.

### 2.7 First-level statistical analysis

Single-subject data were analyzed separately in three spaces: vertexwise for the left and right hemispheres and voxelwise for MNI305 volumetric space (Evans et al., 1993). The first-level analysis used a generalized linear model (GLM) to fit the time course of each participant to a model of the ideal neural response to the stimulus. The six predictors of interest were low, middle and high frequencies in each ear (i.e. left and right). These predictors were convolved with a canonical hemodynamic response function, and the six motion correction parameters were added as nuisance regressors to create the ideal model. In keeping with the study by Liem and colleagues (Liem et al., 2012), we did not model either silence or scanner noise and treated them as part of the baseline. The silent condition was included to vary interstimulus intervals and was treated as baseline to better estimate the overall auditory stimulus-response vs baseline.

A simplified model, showing only the predictors of interest, is shown in Figure 1B.

Statistical maps were created for each participant for the following contrasts:

1. All tones (low, middle, and high frequencies; left and right combined).
2. Low tones (left and right combined).
3. Middle tones (left and right combined).
4. High tones (left and right combined).

### 2.8 Second-level statistical analysis

Initially, statistical maps of all subjects were combined to identify brain regions activated across all frequencies, as well as by each frequency individually. Group differences in activation between CPHIV and control children were then examined using a general linear model in FreeSurfer (*mri_glmfit*). Three additional analyses were conducted: one excluding children with missing hearing data or hearing loss in one or both ears, one excluding left-handed children, and one including sex at birth as a covariate. The decision to control for sex and exclude left-handed children was motivated by evidence that both factors can influence the functional organization of the brain (Andrushko et al., 2023; Nair et al., 2019). Statistical maps were initially examined at a voxelwise threshold of *p* = 0.05. When visualizing activation across all children, this threshold resulted in extensive activation and deactivation in both hemispheres, making the spatial patterns difficult to interpret. Therefore, for visualization purposes, these maps were displayed using a more stringent voxelwise threshold (*p* = 10^−8^). All other statistical maps were thresholded at *p* = 0.05 with clusterwise correction as described below.

To correct for multiple comparisons, clusterwise inference was performed using Monte Carlo simulation as implemented in FreeSurfer's *mri_glmfit-sim* (Forman et al., 1995) across both hemispheres (*- -2spaces*). Clusters were considered significant at a clusterwise corrected α = 0.05. Given the *a priori* directional hypothesis that control children would show greater activation of the auditory cortex than CPHIV, the minimum cluster-size estimation was based on a one-tailed test.

For analyses conducted in MNI305 volumetric space, no directional hypotheses were specified. Accordingly, clusterwise correction was performed across all three spaces (left hemisphere, right hemisphere, and MNI305 space), and both positive and negative statistical values were included, corresponding to a two-tailed test.

### 2.9 Further statistical analysis of FreeSurfer outputs

To examine whether the magnitude of auditory cortex activation varied as a function of tone frequency, frequency-specific activation maps were thresholded at *p* = 10^−8^. For each frequency (low, middle, high), the single largest suprathreshold cluster within each hemisphere's auditory cortex was identified and used to define regions of interest (ROIs). These ROIs were derived from activation maps computed across the full sample of children included in the final analysis, collapsing across diagnostic groups. Mean beta values were then extracted for each participant from these ROIs using the corresponding frequency-specific beta maps (i.e., ROIs defined from low-frequency activation maps were applied to the low-frequency beta images).

Mean beta values were analyzed separately for each hemisphere using one-way repeated-measures ANOVAs (Type III sums of squares). The assumption of sphericity was assessed using Mauchly's test, and Greenhouse-Geisser corrections were applied to the degrees of freedom when this assumption was violated. When the overall ANOVA indicated trend-level significance (*p* ≤ 0.10), Bonferroni-adjusted pairwise *t*-tests were conducted to further examine differences between frequency conditions.

Effect sizes (Cohen's *d*) for between-group differences were estimated using ROIs defined by clusters within the auditory cortex that showed significant group differences in the corresponding whole brain contrast after cluster-level correction for multiple comparisons (voxelwise *p* = 0.05, clusterwise α = 0.05). Mean contrast-specific activation values were extracted from these ROIs for each participant and used to compute Cohen's *d*. These ROIs were used solely for effect size estimation and not for hypothesis testing.

For visualization and non-parametric group comparisons, additional ROIs were defined based on the main effect of auditory stimulation relative to baseline. Specifically, the ROI-defining contrast (see Section 2.7), in which all tone events (low, middle, and high frequency events presented to either ear) were combined into a single regressor, was used to define the ROIs.

**Figure 3:**
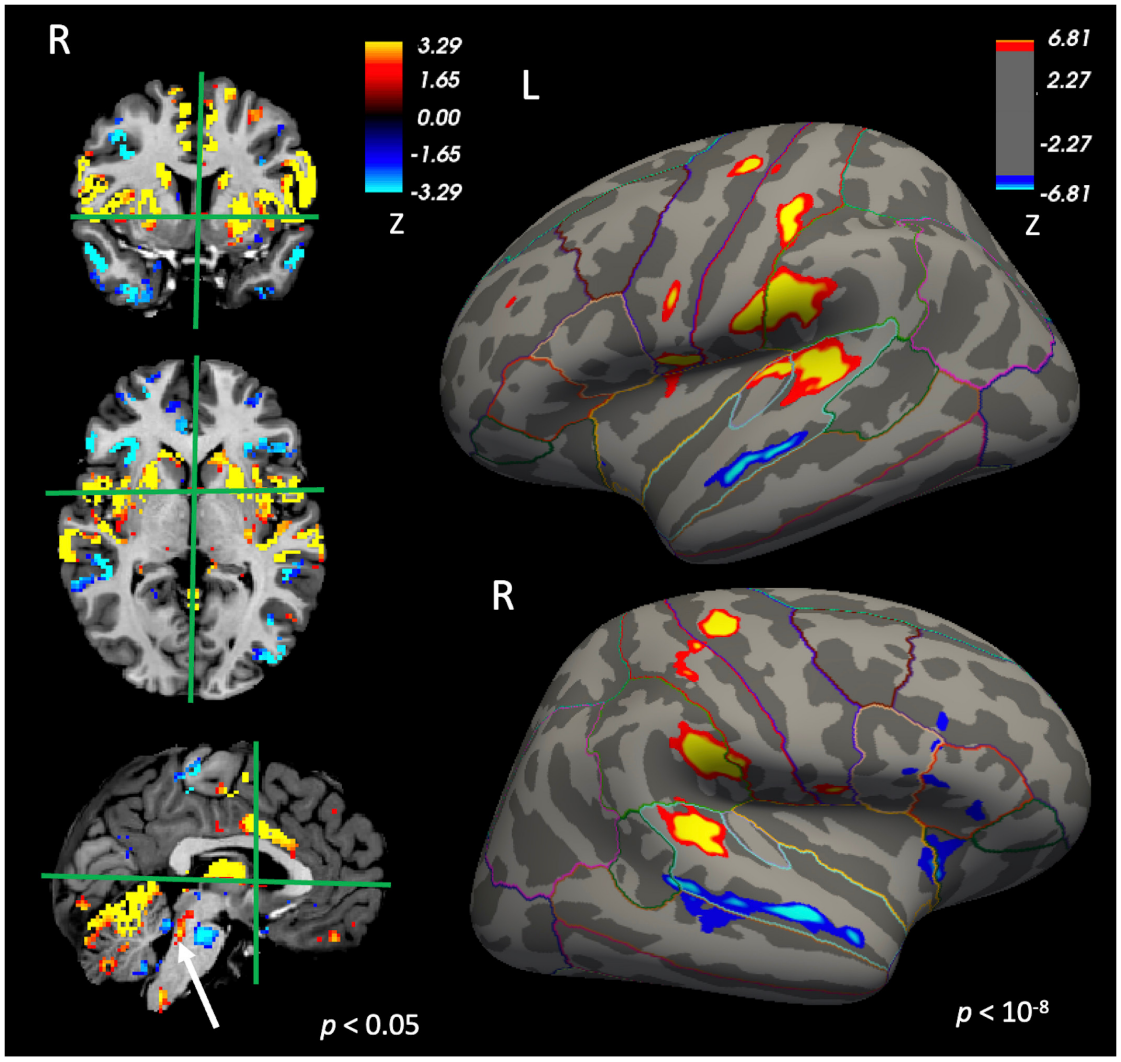
Regions showing significant activation (warm colors) and deactivation (cool colors) across all children during pure-tone perception, collapsed across frequencies. The sagittal view highlights the activation of the inferior colliculus with a white arrow. Lines on the inflated cortical surfaces denote anatomical boundaries defined by FreeSurfer.

At the group level, the beta map representing this main effect across all participants was thresholded at *p* = 10^−8^. Spatially contiguous suprathreshold clusters exceeding the statistical threshold within the bilateral auditory cortices were identified and used to define ROIs (corresponding to the auditory cortex regions shown in Figure 3).

Importantly, group membership and all group-related contrasts were not used in ROI selection. In the group-level GLM, task and group regressors were specified separately and were orthogonal. Thus, the ROI-defining contrast was statistically independent of subsequent between-group comparisons. Although defining ROIs from activation maps collapsed across groups may spatially reflect the dominant activation pattern in the combined sample, voxel selection was based solely on the overall task activation contrast and not on any statistic reflecting group differences, thereby avoiding circularity (Kriegeskorte et al., 2009).

Mean activation values were then extracted from these ROIs for each participant for each frequency condition and for all tones combined. Between-group comparisons (control vs. CPHIV) were performed using two-sample Wilcoxon rank-sum tests.

To examine associations between auditory cortex activation and behavioral measures of hearing thresholds, mean activation values were extracted from the bilateral auditory cortex ROIs defined by the main effect of all tones versus baseline (see above). For each participant, mean activation values were obtained from these ROIs separately for each hemisphere and frequency condition and correlated with hearing thresholds at the corresponding frequencies. Because hearing thresholds at 1,500 Hz were not directly measured, they were estimated for each ear as the average of the 1,000 Hz and 2,000 Hz thresholds. Thresholds were then averaged across ears to obtain a single hearing value per frequency per child. As hearing thresholds were not normally distributed, both the thresholds and activation values were rank-transformed across all children with valid hearing data. Pearson correlation coefficients were computed on the ranked data separately for the control and CPHIV groups.

## 3 Results

After exclusions, we report results from 108 participants: 48 controls and 60 CPHIV. Exclusions were due to motion (*n* = 6), poor quality functional data (*n* = 16), poor quality structural data (*n* = 2), missing functional data (*n* = 1), functional log errors (*n* = 3), and structural abnormalities (very large ventricles; *n* = 1). The proportion of excluded children was similar in CPHIV (23.1%) and control children (18.6%) (χ^2^= 0.18, *p* = 0.676). Sample characteristics are shown in Table 1. CPHIV did not significantly differ from controls in sex at birth (χ^2^ = 1.73, *p* = 0.189), handedness (χ^2^ = 0.00, *p* > 0.999), monthly household income (*p* = 0.465), or age (*p* = 0.451). The groups did differ significantly in home language (*p* = 0.016) and approached significance regarding caregiver's education (*p* = 0.058). The control group included 27 children who are HIV-unexposed and uninfected (CHUU) and 21 children perinatally HIV-exposed but uninfected (CHEU).

**Table 1:** Sample characteristics.

Characteristic	Control *n* = 48	CPHIV *n* = 60	*p*
Demographics
Sex at birth: female	21 (43.75%)	35 (58.33%)	0.189^c^
Age at scan (years)^a^	11.57 (0.23)	11.60 (0.26)	0.451^d^
Hand preference: right	42 (87.50%)	53 (88.33%)	>0.999^c^
**Home language^1^**			**0.016^e^**
Xhosa	35 (74.47%)	56 (93.33%)	
Afrikaans	10 (21.28%)	3 (5.00%)	
English	2 (4.25%)	1 (1.67%)	
Socioeconomic			
**Caregiver education – highest level attained^2^**			0.058^e^
Some primary school	2 (4.17%)	11 (18.64%)	
Completed primary school	2 (4.17%)	3 (5.08%)	
Some high school	30 (62.50%)	26 (44.07%)	
Completed high school	8 (16.67%)	15 (25.42%)	
>High school	6 (12.50%)	4 (6.78%)	
**Monthly household income^2^**			0.465^e^
<R1,000	2 (4.17%)	3 (5.08%)	
R1,001–R1,500	7 (14.58%)	6 (10.17%)	
R1,501–R2,500	8 (16.67%)	20 (33.90%)	
R2,501–R5,700	21 (43.75%)	22 (37.29%)	
R5,701–R10,000	8 (16.67%)	7 (11.86%)	
R10,001–R15,000	1 (2.08%)	1 (1.69%)	
>R20,000	1 (2.08%)	0 (0.00%)	
Clinical (CPHIV only)			
Age at first VL suppression (weeks)^b^^, 3^		42.64 (32.39, 81.46)	
Age ART initiation (weeks)^b^^, 3^		9.43 (7.43, 12.00)	
Continuous ART^2^		27 (46.55%)	
Enrollment			
**Viral load^2^**			
Low (400–750,000 RNA copies/ml)		25 (43.10%)	
High (>750,000 RNA copies/ml)		33 (56.90%)	
**CD4 count (cells/mm^3^)^b^^, 3^**		1,563.00 (1,166.75, 2,227.75)	
**CD4 percent^a^^, 2^**		33.69 (10.88)	
11 years			
**Viral load^3^**			
Suppressed (< 400 RNA copies/ml)		57 (96.61%)	
Low (400–750,000 RNA copies/ml)		2 (3.39%)	
**CD4 count (cells/mm^3^)^b^^, 2^**		856.00 (628.00, 994.00)	
**CD4 percent^a^^, 3^**		38.55 (7.38)	
**CD8 count (cells/mm^3^)^a^^, 2^**		735.73 (276.26)	
**CD8 percent^a^^,^^4^**		32.27 (7.30)	

Values are n (%);

^a^Mean (SD);

^b^Median (Q1, Q3).

Tests used:

^c^Pearson's Chi-squared test with Yates's continuity correction;

^d^Welch Two-sample t-test;

^e^Fisher's exact test.

R, South African rand; VL, plasma viral load (RNA copies/ml); ART, antiretroviral therapy.

^1^One control child speaking English and Afrikaans at home was excluded. Missing data for ^2^1 CPHIV, ^3^2 CPHIV.

Statistically significant *p*-values (*p* ≤ 0.05) are shown in bold.

All CPHIV had initiated ART before 18 months of age (age range 5.9–75.7 weeks). The first-line ART regimen for all CHER participants when starting ART consisted of Zidovudine (ZDV), C Lamivudine (3TC), and C Lopinavir-Ritonavir (LPV/r, KaletraR) (Violari et al., 2008; Cotton et al., 2013). At the time of the MRI scan, 46 (77%) of the children whose imaging results were included in this study were still on ZDV, 3TC, and LPV/r; 7 were on Abacavir (ABC), 3TC, and LPV/r; 4 on ABC, 3TC, and Efavirenz (EFV); and 3 were on a combination of other drugs.

### 3.1 Hearing results

Comparison between the hearing data of the control and CPHIV groups is shown in Table 2. Across most frequencies, children with perinatally acquired HIV (CPHIV) showed slightly higher hearing thresholds than controls (i.e., poorer hearing sensitivity). In the right ear, thresholds were significantly higher in the CPHIV group at 500 Hz (median = 10 dB vs. 5 dB; *p* = 0.004) and 1,000 Hz (median = 10 dB vs. 5 dB; *p* = 0.046), but not at 2,000 or 4,000 Hz (both *p* > 0.26). In the left ear, thresholds were also significantly higher in the CPHIV group at 500 Hz (*p* = 0.043), 1,000 Hz (*p* = 0.009), and 4,000 Hz (*p* = 0.040), but not at 2,000 Hz (*p* = 0.103).

**Table 2:** Hearing thresholds and hearing loss in control and CPHIV groups.

	Control					CPHIV					
Hearing threshold	*n* ^*^	Median	Q1	Q3	Range	*n* ^*^	Median	Q1	Q3	Range	*p* ^a^
Right 500 Hz	47	5	0	10	−5–20	57	10	5	10	0–95	**0.004**
Right 1,000 Hz	46	5	5	10	−5–15	57	10	5	10	10–105	**0.046**
Right 2,000 Hz	47	5	5	10	0–25	57	5	5	10	−5–115	0.642
Right 4,000 Hz	47	5	5	10	−5–45	57	10	5	10	0–120	0.264
Left 500 Hz	47	5	0	10	−10–15	56	5	5	10	−5–50	**0.043**
Left 1,000 Hz	46	5	0	9	−5–25	57	10	5	10	0–55	**0.009**
Left 2,000 Hz	47	5	0	10	0–25	57	5	5	10	0–35	0.103
Left 4,000 Hz	47	5	0	10	−5–50	56	10	5	15	0–20	**0.040**
Hearing loss	*n* (%)					***n*** **(%)**					***p** ^*b*^*
Both ears	2 (4.26)					1 (1.75)					0.588
Left ear only	0 (0.00)					3 (5.26)					0.250
Right ear only	0 (0.00)					1 (1.75)					1.000
No hearing loss	45 (95.74)					52 (91.23)					0.453

^*^n varies due to missing hearing data for some children.

^a^Wilcoxon rank-sum test;

^b^Fisher's exact test; significant values in bold.

CPHIV, children with perinatally acquired HIV; Q, quartile.

Pure-tone average for each side is the average hearing threshold in that ear over the four frequencies. A higher threshold means worse hearing.

Hearing loss in each ear is defined as having a pure-tone average of 500, 1,000, 2,000, and 4,000 Hz in that ear greater than 15 dB.

Hearing data were completely missing for four children (control = 1, CPHIV = 3).

Based on the pure-tone average (PTA) across 500–4,000 Hz, the prevalence of hearing loss did not significantly differ between groups. In the control group, 4.3% had bilateral hearing loss, and none had unilateral loss. In the CPHIV group, 1.8% had bilateral loss, 5.3% had left-ear loss, and 1.8% had right-ear loss; none of these proportions were significantly different from controls (all *p* ≥ 0.25). Most children in both groups had normal hearing (95.7% controls; 91.2% CPHIV).

### 3.2 fMRI results for all children

The children performed well on the auditory task in the scanner, achieving a mean accuracy of 86.35%. The performance did not differ between CPHIV (86.27%) and control children (86.46%) (*p* > 0.9).

Figure 3 shows regions exhibiting significant responses across all children during tone perception, collapsed across frequencies. At a vertexwise uncorrected threshold of *p* < 10^−8^, robust activation was observed bilaterally along the superior temporal gyrus, encompassing the auditory cortex, as well as in precentral (motor) and supramarginal regions. In addition, both hemispheres exhibited deactivation in superior temporal regions located inferior to the PACs.

In volumetric MNI305 space, overall activation was weaker. To visualize smaller regions potentially involved in auditory processing, results are displayed at a voxelwise uncorrected threshold of *p* = 0.05. At this threshold, activation was widespread, with a prominent bilateral cluster in the cerebellum. Notably, activation was also observed in the region of the inferior colliculus (white arrow), a key component of the CAS, although this effect did not survive cluster-level correction.

Clusters showing activation or deactivation in response to auditory stimulation that survived cluster-level correction are summarized in Table 3 (vertexwise *p* = 10^−8^, clusterwise α = 0.05 for surface-based analyses; voxelwise *p* = 0.05, clusterwise α = 0.05 for volumetric analyses in MNI305 space).

**Table 3:** Regions showing significant activation (deactivation) in all children (CPHIV and controls) when hearing pure tones at low, middle, and high frequencies.

Region	Size (mm^2^)	Peak MNI co-ordinates (mm)		
		x	y	z
Left cortical hemisphere				
Superior temporal	703.34	−53.4	−33.7	7.8
Supramarginal	671.36	−58.9	−25.8	22.0
Superior frontal	440.55	−8.3	−1.0	52.3
Precentral	283.93	−48.9	−2.0	8.0
Postcentral	281.00	−51.8	−24.2	47.7
(Superior temporal)	(266.49)	−51.9	−12.3	−11.5
Precentral	193.51	−37.6	−19.7	56.6
Precentral	54.43	−58.3	3.5	27.0
Postcentral	16.23	−38.9	−27.1	48.8
Rostral middle frontal	12.34	−39.5	38.0	27.1
(Insula)	(2.32)	−29.5	16.1	−6.9
(Superior parietal)	(0.52)	−19.0	−82.0	39.8
Right cortical hemisphere				
(Superior temporal)	(591.19)	53.9	−6.4	−11.6
Supramarginal	429.81	56.5	−21.1	21.4
Superior temporal	423.16	57.8	−30.3	8.3
Precentral	294.31	39.0	−14.7	58.8
Postcentral	202.86	40.4	−22.8	47.2
Superior frontal	181.78	10.1	−1.7	54.7
(Lateral orbitofrontal)	(129.06)	28.5	20.0	−5.9
Precentral	104.50	48.4	2.9	7.6
(Pars triangularis)	(53.25)	52.1	21.9	9.8
(Pars triangularis)	(46.15)	43.6	31.6	−0.7
(Rostral middle frontal)	(22.00)	42.2	27.6	21.5
(Pars opercularis)	(19.74)	47.9	25.9	17.7
(Pars opercularis)	(17.37)	55.0	17.7	14.9
(Precuneus)	(8.09)	15.6	−69.6	38.6
(Precuneus)	(4.14)	11.4	−66.7	36.5
Superior frontal	3.43	9.4	−0.9	67.2
Superior frontal	1.57	7.4	−1.3	64.7
MNI305 space				
Right cerebellum cortex	18,353.00	24.0	−55.0	−21.0

Left and right hemispheres thresholded at voxelwise *p* = 10^−8^, clusterwise α = 0.05. MNI305 regions were thresholded at voxelwise *p* = 0.05, clusterwise α = 0.05. All clusters listed have clusterwise *p* < 0.003.

**Figure 4:**
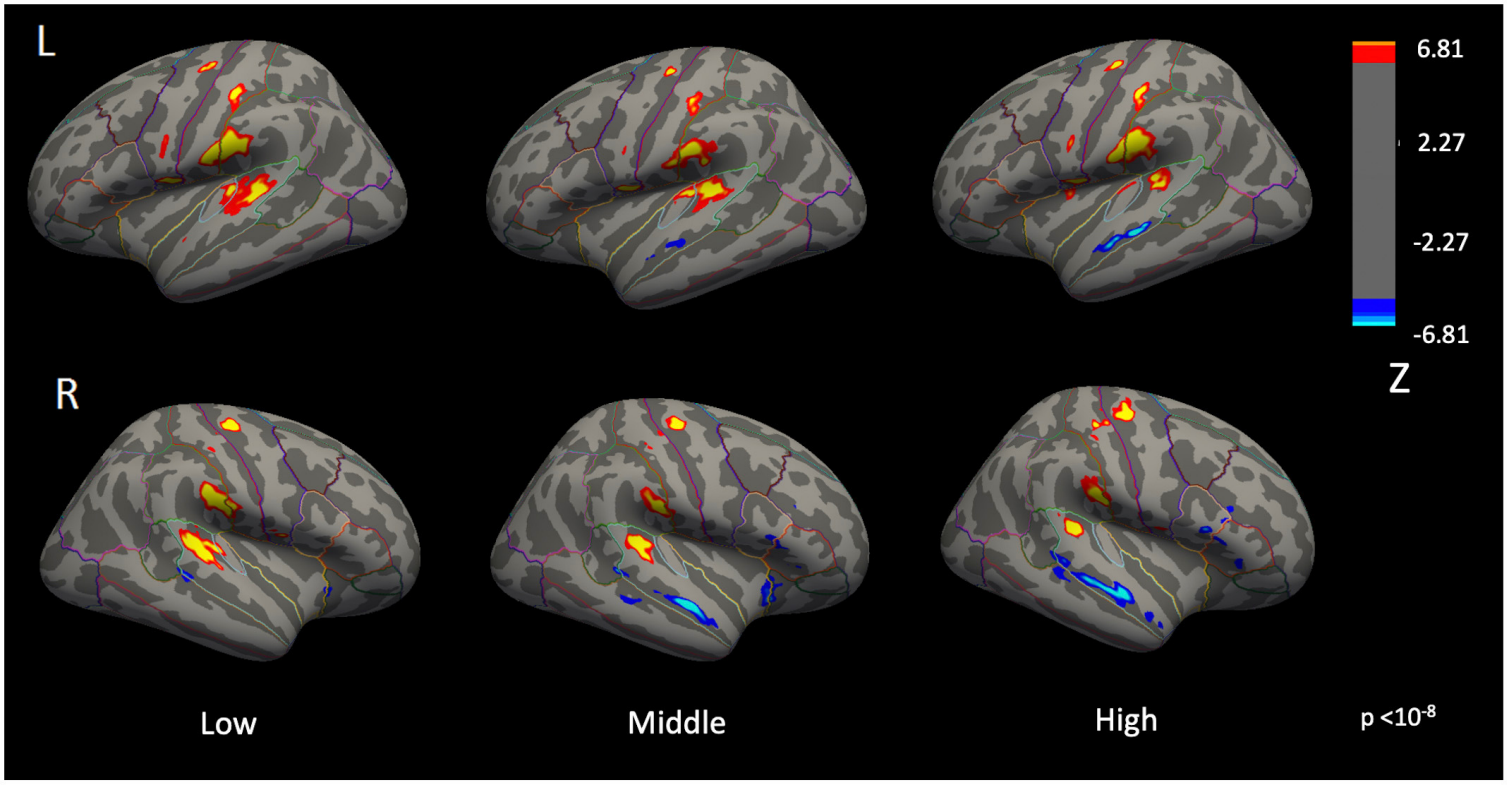
Cortical activation patterns during the pure-tone perception at 500Hz (low), 1,500Hz (middle), and 4,000Hz (high) frequencies (vertexwise *p* < 10^−8^). As the stimulus frequency increases, the spatial extent of auditory cortex activation decreases.

Figure 4 illustrates differences in auditory cortex activation while listening to pure tones of low, middle, and high frequencies (vertexwise *p* = 10^−8^). As frequency increased, the extent of activation in the auditory cortex decreased. In the right hemisphere, regions of activation were 467.06 mm^2^, 299.49 mm^2^, and 169.96 mm^2^ for 500 Hz, 1,500 Hz, and 4,000 Hz, respectively. Corresponding values for the left hemisphere were 704.12 mm^2^, 629.98 mm^2^, and 169.96 mm^2^. Conversely, as frequency increased, the extent of deactivations in the inferior portions of the superior temporal regions also increased.

To assess whether increasing stimulus frequency was associated with changes in activation magnitude, beyond differences in spatial extent, mean activation values were extracted from frequency-specific left and right auditory cortex ROIs for each participant. In both hemispheres, mean activation decreased monotonically with increasing stimulus frequency. In the right PAC, mean activation values were 0.245, 0.219, and 0.194 at 500 Hz, 1,500 Hz, and 4,000 Hz, respectively. Corresponding values in the left PAC were 0.208, 0.178, and 0.163.

A repeated-measures ANOVA revealed a significant main effect of stimulus frequency in the right auditory cortex ROIs [*F*_(2, 214)_ = 3.57, *p* = 0.030]. Bonferroni-corrected *post-hoc* pairwise comparisons indicated that only the contrast between 500 Hz and 4,000 Hz remained significant (*p* = 0.034). In the left auditory cortex ROIs, the effect of frequency approached but did not reach statistical significance after Greenhouse-Geisser correction [*F*_(1.86, 199.18)_ = 2.19, *p* = 0.056], and no *post-hoc* pairwise comparisons were significant.

### 3.3 Control children vs. CPHIV

Auditory responses to pure tones were compared between control children and CPHIV. A Wilcoxon rank-sum test indicated no significant differences in head motion between groups, either for average volume-wise motion (median = 0.123 for controls, 0.125 for CPHIV) or for maximum pairwise displacement (median = 1.051 for controls, 1.167 for CPHIV), both *p* > 0.7.

**Figure 5:**
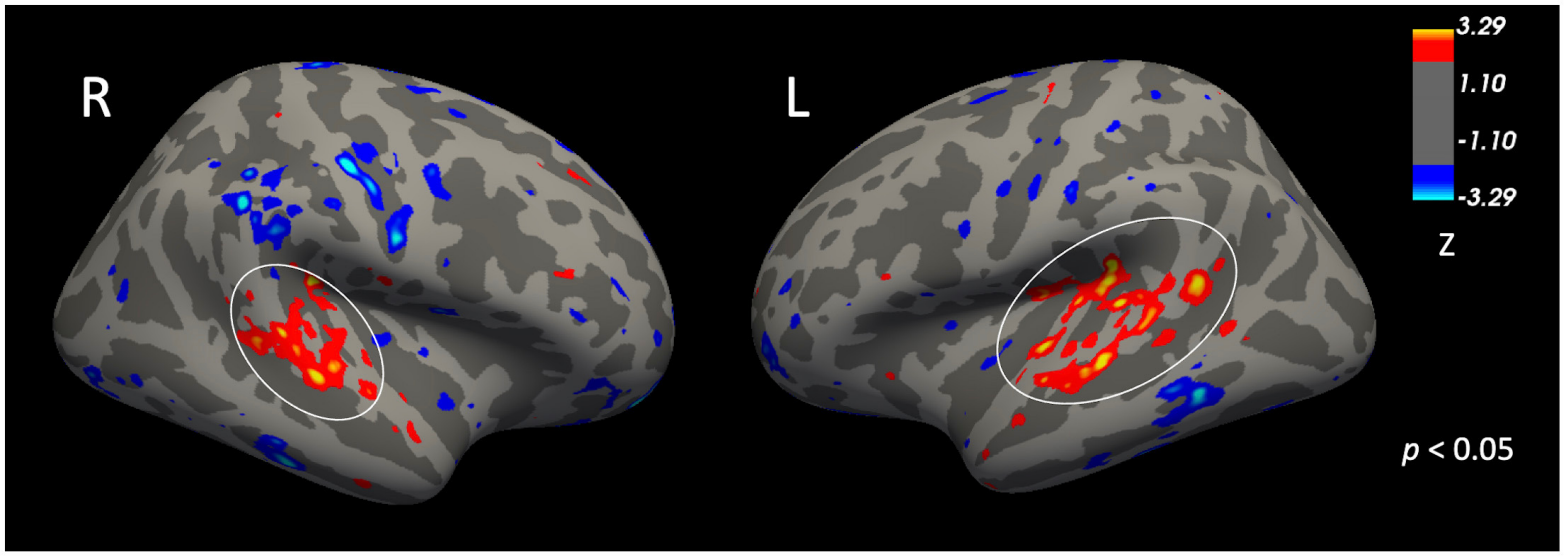
Clusters within the bilateral auditory cortices showing greater activation in control children than in CPHIV during pure-tone perception (*p* < 0.05). Peak coordinates, cluster size, and effect sizes are reported for left (MNI 42.8, −34.1, 18.0; area = 2,221.45 mm^2^; Cohen's *d* = 0.634) and right (MNI 39.8, −31.2, 22.3; area = 1,314.89 mm^2^; Cohen's *d* = 0.641) hemispheres. Circled regions survive cluster-level correction (α = 0.05). Warm colors indicate greater activation in controls; cool colors indicate greater activation in CPHIV.

Figure 5 shows the group comparison of auditory cortex activation collapsed across all frequencies (all tones > baseline). Following cluster-level correction (vertexwise *p* = 0.05, clusterwise α = 0.05), CPHIV exhibited significantly reduced activation in bilateral auditory cortex relative to controls. The significant clusters measured 2,221.45 mm^2^ in the left hemisphere and 1,314.89 mm^2^ in the right hemisphere, with peak MNI coordinates at −42.8, −34.1, 18.0 (left) and 39.8, −31.2, 22.3 (right). Effect sizes were in the medium-to-large range (Cohen's *d* = 0.634 for the left hemisphere and 0.641 for the right), indicating substantial differences in the strength of auditory cortex responses between groups.

These findings remained robust after excluding children without hearing data (1 control; 3 CPHIV) and those with hearing loss in one or both ears (2 controls; 5 CPHIV). Under these restrictions, effect sizes increased to *d* = 0.741 (left) and *d* = 0.770 (right), approaching the large range. Group differences were also essentially unchanged when controlling for sex and after excluding left-handed participants.

**Figure 6:**
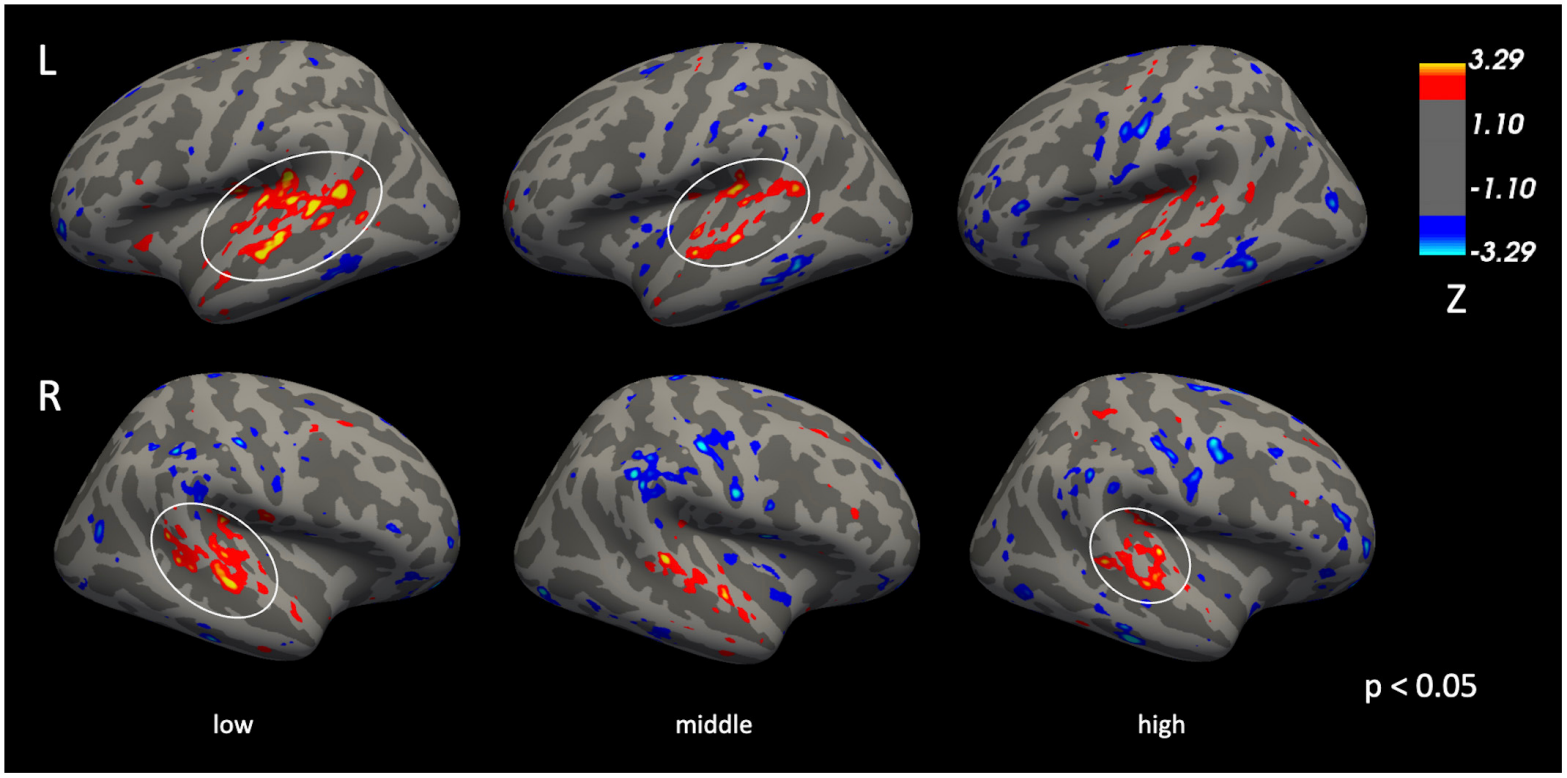
Group differences in activation during the pure-tone perception at low (500 Hz), middle (1,500 Hz), and high (4,000 Hz) frequencies. Regions within the auditory cortex show greater activation in control children than in CPHIV at *p* < 0.05, although only circled regions survive cluster-level correction (α = 0.05). Warm colors indicate greater activation in controls; cool colors indicate greater activation in CPHIV.

When frequencies were examined separately (Figure 6), group differences in auditory cortex activation were observed at all frequencies and in both hemispheres (vertexwise *p* = 0.05, clusterwise α = 0.05). In the left hemisphere, the spatial extent of the cluster showing reduced activation in CPHIV decreased with increasing stimulus frequency.

At 500 Hz, significant bilateral clusters survived cluster-level correction, with a left hemisphere cluster of 2,666.58 mm^2^ (peak MNI −42.3, −43.6, 18.3; *d* = 0.711) and a right hemisphere cluster of 1,242.88 mm^2^ (peak MNI 54.6, −22.0, −2.0; *d* = 0.629). At 1,500 Hz, a significant cluster was observed only in the left hemisphere (972.79 mm^2^; peak MNI −55.7, −28.2, −1.6; *d* = 0.601). At 4,000 Hz, significance was limited to the right hemisphere (849.43 mm^2^; peak MNI 46.9, −22.0, 7.7; *d* = 0.630).

**Figure 7:**
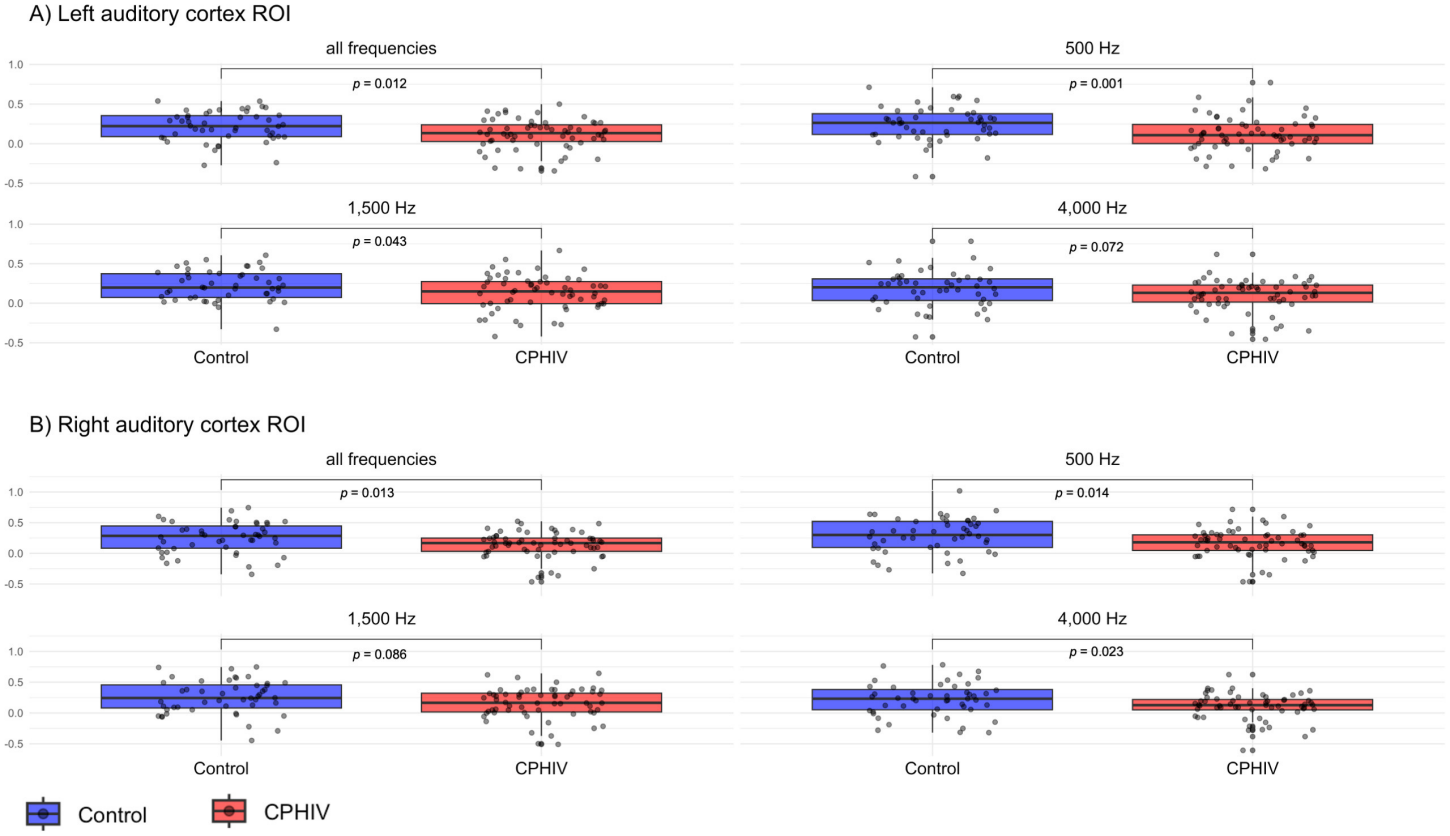
Group differences in mean activation within left and right auditory cortex ROIs defined based on the main effect of all tones relative to baseline across all children. **(A)** Mean activation extracted from the left auditory cortex ROI for all frequencies combined and separately for 500 Hz, 1,500 Hz, and 4,000 Hz. **(B)** Mean activation extracted from the right auditory cortex ROI for the same conditions. Boxplots display the distribution of individual activation values for the control (blue) and CPHIV (red) groups; gray points represent individual participants. Boxes show the median and interquartile range (Q1–Q3), with whiskers extending to values within 1.5 x IQR. *p*-*values* reflect Wilcoxon rank-sum tests. Across hemispheres, CPHIV consistently show reduced auditory cortex activation, with the largest differences observed at 500 Hz and in the combined-frequency condition.

Figure 7 illustrates the distributions of mean activation values from the left and right auditory cortex ROIs defined from the main effect of all tones > baseline. Across conditions, CPHIV exhibited lower activation than control children. In the left hemisphere, activation was significantly reduced in CPHIV for all frequencies combined (*p* = 0.012), 500 Hz (*p* = 0.001), and 1,500 Hz (*p* = 0.043), with a trend toward reduced activation at 4,000 Hz (*p* = 0.072). A similar pattern was observed in the right hemisphere, with significant group differences for all frequencies combined (*p* = 0.013), 500 Hz (*p* = 0.014), and 4,000 Hz (*p* = 0.023), and a trend-level difference at 1,500 Hz (*p* = 0.086).

Pearson correlations between ranked hearing thresholds and ranked mean activation values extracted from the bilateral auditory cortex ROIs defined from the main effect of all tones > baseline are presented in Table 4. Across most frequencies, correlations were not significant (all *p* > 0.35) and did not show a consistent pattern.

**Table 4:** Correlation (95% CI) between pure-tone threshold and mean activation.

Frequency	Control					CPHIV				
		LH		RH			LH		RH	
	*n* ^*^	Correlation	*p*	Correlation	*p*	*n* ^*^	Correlation	*p*	Correlation	*p*
500 Hz	47	−0.01 (−0.29, 0.28)	0.961	0.02 (−0.27, 0.31)	0.892	56	0.13 (−0.14, 0.38)	0.357	0.09 (−0.18, 0.35)	0.504
1,500 Hz	46	0.02 (−0.27, 0.31)	0.906	−0.04 (−0.33, 0.25)	0.792	57	−0.13 (−0.37, 0.14)	0.352	−0.03 (−0.29, 0.23)	0.802
4,000 Hz	47	0.00 (−0.29, 0.28)	0.975	−0.11 (−0.39, 0.18)	0.460	56	0.42 (0.17, 0.61)	**0.001**	0.29 (0.03, 0.51)	**0.030**

Correlation, Pearson correlations of ranked variables, ranked over all subjects with valid values; CI, confidence interval; LH, left hemisphere; RH, right hemisphere; CPHIV, Children with perinatally acquired HIV; pure-tone threshold at each frequency is the average over the thresholds of the left and right ears at that frequency; pure-tone threshold at 1,500 Hz was estimated by averaging between the hearing thresholds at 1,000 Hz and 2,000 Hz; ^*^n varies due to missing hearing data for some children; significant *p*-values in bold.

**Figure 8:**
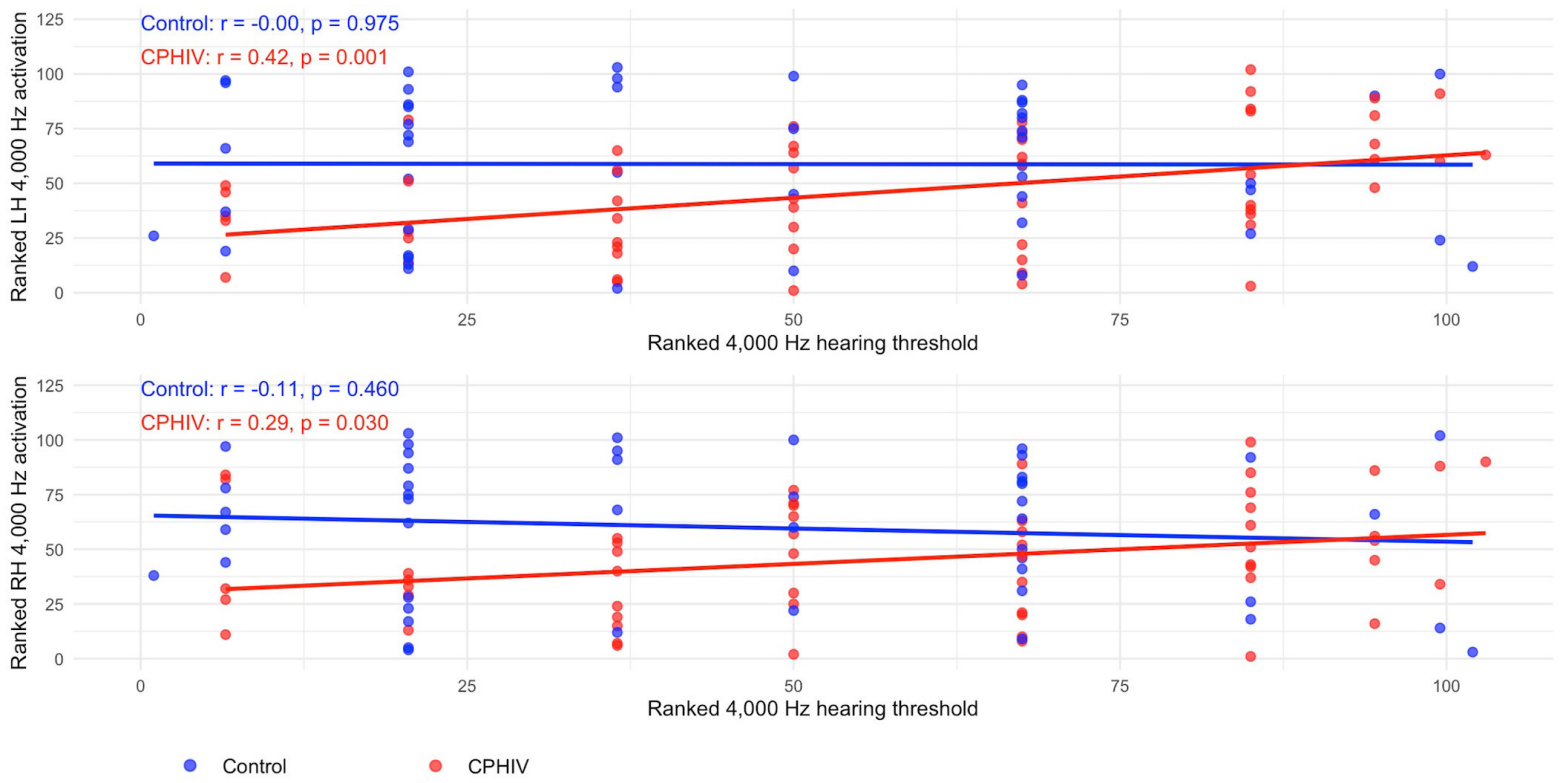
Scatterplots illustrating the relationship between ranked 4,000 Hz hearing thresholds and ranked mean auditory cortex activation during the perception of 4,000 Hz tones. The top panel shows the left hemisphere and the bottom panel shows the right hemisphere. Blue points and regression lines represent control children; red points and regression lines represent CPHIV. Variables were rank transformed due to non-normal distributions. In CPHIV, poorer hearing (higher thresholds) was associated with greater auditory cortex activation, whereas no clear relationship was observed in controls. LH, left hemisphere; RH, right hemisphere; CPHIV, children with perinatally acquired HIV.

A notable exception emerged in CPHIV at 4,000 Hz. In this group, higher (poorer) hearing thresholds were positively correlated with mean activation in both the left (*r* = 0.42, *p* = 0.001) and right (*r* = 0.29, *p*= 0.030) auditory cortices (Figure 8). Thus, among CPHIV, poorer high-frequency hearing sensitivity was associated with stronger bilateral auditory cortex responses to 4,000 Hz tones.

Overall, the pattern of results indicates broadly reduced bilateral auditory cortex activation in CPHIV, with the most pronounced group differences at lower frequencies and a selective high-frequency association between poorer hearing and greater auditory cortex activation.

## 4 Discussion

To our knowledge, this study is the first to use fMRI to compare CAS responses to pure-tone stimuli between CPHIV and control children. Control children and CPHIV were scanned while listening to monaural pure tones of varying frequencies to investigate potential HIV-related differences in auditory processing. We found that, despite having largely normal peripheral hearing, CPHIV receiving ART exhibited reduced bilateral auditory cortex responses compared with control children.

### 4.1 Responses across all children

Auditory stimulation is known to elicit robust bilateral activation of the auditory cortices (Dhankhar et al., 1997; Grady et al., 1997; Jäncke et al., 1998; Binder et al., 2000), and our findings are consistent with this literature. Across all participants, pure tones at 500 Hz, 1,500 Hz, and 4,000 Hz reliably engaged bilateral auditory cortex regions (Figure 3). In addition, bilateral activation was observed in the precentral (motor) cortex, as expected given the motor response required by the task.

Beyond cortical regions, activation was also detected in the inferior colliculus, a key relay within the auditory pathway (Du et al., 2025), although this effect did not survive correction for multiple comparisons. Given the small size of the inferior colliculus, the absence of cluster-level significance is not unexpected.

Because the auditory cortex is organized tonotopically, we expected different sound frequencies to preferentially engage distinct subregions of the auditory cortex. Clear tonotopic separation was not observed, likely because the differences between the selected frequencies were too small to be reliably resolved at the spatial resolution of the present fMRI data. Instead, we observed a frequency-dependent difference in the spatial extent of auditory cortex activation, with lower-frequency tones eliciting more spatially extensive activation than higher-frequency tones (Figure 4). This pattern is consistent with prior findings by Bilecen et al. (1998), who reported a larger extent of auditory cortex activation in response to 500 Hz tones compared with 4,000 Hz tones in most participants.

One plausible explanation for this frequency-dependent difference in activation extent is that lower-frequency sounds, which have longer wavelengths, produce broader displacement of the basilar membrane within the cochlea. This broader mechanical response may stimulate a larger population of hair cells, ultimately leading to engagement of a wider region of the auditory cortex. In parallel with these spatial effects, mean activation magnitude within frequency-specific auditory cortex ROIs tended to decrease with increasing frequency, although most differences did not reach statistical significance. Together, these findings suggest that lower-frequency tones elicit both more spatially extensive and stronger cortical responses than higher-frequency tones.

### 4.2 CPHIV vs. control children

CPHIV exhibited modestly elevated hearing thresholds at several frequencies compared with children without HIV, with the largest differences observed at lower frequencies. Although these differences were small in magnitude (typically about 5 dB), they were statistically significant at multiple frequencies in both ears. Importantly, despite these threshold differences, the overall prevalence of clinically defined hearing loss—based on the pure-tone average across 500–4,000 Hz—did not differ between groups, and the majority of children in both groups demonstrated hearing thresholds within the normal range.

The central finding of this study is that CPHIV receiving ART exhibited reduced bilateral auditory cortex responses while listening to pure tones relative to controls. These group differences were spatially extensive and associated with medium to large effect sizes across hemispheres and frequencies. Notably, effect sizes increased after excluding children with hearing loss, supporting the interpretation that these differences reflect alterations in central auditory processing rather than differences in peripheral hearing ability.

It should be noted, however, that effect sizes were calculated within ROIs derived from group difference maps. As such, these estimates are not statistically independent of the contrasts used to define the ROIs and may therefore be inflated (Kriegeskorte et al., 2009; Vul et al., 2009). Accordingly, these effect sizes should be interpreted as descriptive indices of magnitude rather than unbiased estimates of the true population effect. Importantly, the presence and spatial extent of the group differences were established using whole brain corrected analyses, independent of the ROI-based effect size estimation.

Group differences remained robust after controlling for sex, excluding left-handed participants, and accounting for head motion, indicating that these factors are unlikely to explain the observed effects. Reduced auditory cortex responses in CPHIV were evident across all tone frequencies, suggesting a generalized reduction of auditory cortex responsiveness rather than frequency-specific alterations. Although both the spatial extent and magnitude of group differences decreased with increasing frequency, the direction and magnitude of the effect remained consistent. This pattern suggests globally reduced neural responsiveness within auditory cortices rather than selective impairment of specific frequency channels. These differences were observed despite early ART initiation (median age 9.4 weeks) and viral suppression in 97% of the children at the time of neuroimaging, indicating that altered cortical processing persists even in the context of effective long-term treatment.

Across most frequencies, associations between hearing thresholds and mean auditory cortex activation were weak and not significant, indicating no straightforward relationship between peripheral hearing sensitivity and cortical response. A notable exception was observed at 4,000 Hz in CPHIV, where poorer hearing thresholds were associated with stronger activation in both the left and right auditory cortices. This pattern suggests increased recruitment of cortical resources in children with poorer high-frequency hearing, consistent with a compensatory neural response. Overall, these findings point to subtle but consistent differences in auditory cortex activation in CPHIV, with evidence of compensation emerging selectively at higher frequencies.

When considered alongside previous findings from the ARCH cohort, a coherent picture of auditory system involvement in CPHIV begins to emerge.

Earlier work demonstrated elevated minimum detectable sound levels in the poorer ear of CPHIV compared with control children (Torre III et al., 2015a), indicating reduced auditory sensitivity. A critical question is whether this reduced sensitivity reflects peripheral auditory pathology or alterations within central auditory processing. Peripheral dysfunction could reduce afferent input to the auditory cortices, potentially leading to weaker cortical responses even in the absence of primary cortical abnormalities.

The auditory pathway encompasses the outer ear, middle ear, cochlea, vestibulocochlear nerve, and the central auditory system. In the present study, children were included only if there was no evidence of middle ear pathology, as indicated by the absence of otorrhea and normal tympanometric findings (Type A tympanogram with acoustic reflexes present). This minimizes the likelihood that group differences are attributable to impaired middle ear sound transmission, narrowing potential mechanisms to the cochlea, vestibulocochlear nerve, or central auditory system.

Measures of cochlear outer hair cell function in this cohort further inform the interpretation of the present findings. DPOAEs, which index cochlear outer hair cell integrity (Abdala and Visser-Dumont, 2001), were assessed in a subsample of these participants and were not associated with HIV status (Torre III et al., 2015a), consistent with earlier reports (Torre III et al., 2015b). Although this does not exclude all forms of inner ear involvement, these results argue against substantial hair cell dysfunction as a primary driver of the reduced auditory cortex responses in CPHIV observed here, thereby strengthening the case for alterations at more central levels of the auditory system.

ABRs, which index neural conduction along the auditory nerve and lower brainstem (Eggermont, 2019), provide additional insight. Preliminary analyses in this cohort revealed no significant group differences in ABR peak latencies or amplitudes between CPHIV and controls (Torre III et al., 2019), suggesting preserved vestibulocochlear nerve and brainstem function. Importantly, the absence of ABR differences indicates that both groups received comparable auditory input at early stages of the pathway. Together, these findings support the interpretation that the reduced auditory cortex responses observed in CPHIV are unlikely to be driven by peripheral or brainstem dysfunction and instead reflect alterations within the central auditory system.

### 4.3 Alternative explanations for reduced activation

Several alternative mechanisms could contribute to the reduced auditory cortex activation observed in CPHIV. One possibility is long-term exposure to ART, as ART can cause hearing impairment (Marra et al., 1997; Simdon et al., 2001). Because all CPHIV in the present study started ART early in life and remained on treatment throughout childhood, it is not possible to disentangle the effects of HIV infection from those of prolonged ART exposure. Thus, the observed differences may reflect the combined impact of HIV and long-term ART on neural development rather than the effect of the virus alone.

Nutritional factors may also influence auditory system development (Zuanetti et al., 2014; Jain et al., 2025). CPHIV are at increased risk of early malnutrition (Hendricks et al., 2007), which has been associated with long-term neurodevelopmental alterations (Kirolos et al., 2022). Although the groups in the present study did not differ significantly in household income and were drawn from similar communities, detailed nutritional histories were not available.

In addition, early life co-infections and chronic immune activation, which are more prevalent in CPHIV, may influence neurodevelopment through inflammatory or neuroimmune mechanisms (Blokhuis et al., 2019; Wedderburn et al., 2019; Williams et al., 2021). While our cohort was clinically stable at the time of scanning, early life infectious burden and inflammation could contribute to long-term alterations in central auditory processing (Khavarghazalani et al., 2025).

Despite these alternative explanations, several aspects of the present findings are consistent with the involvement of the central auditory system. All children were screened for middle ear pathology, and prior work in this cohort has demonstrated intact cochlear function and normal auditory brainstem responses, making it unlikely that peripheral and brainstem factors alone account for the observed differences in auditory cortex activation. Taken together, these results suggest that cortical-level auditory responses contribute to the auditory differences observed in CPHIV, even though the underlying mechanisms are likely multifactorial.

### 4.4 Study limitations

One limitation of the present study is the absence of an ART-naïve group of CPHIV. Inclusion of such a group would have enabled clearer separation of effects related to HIV infection from those associated with long-term ART exposure. However, in the context of current clinical guidelines that prioritize early initiation of ART in CPHIV, the availability of an ART-naïve comparison group is increasingly rare and ethically constrained.

A further limitation is that the tone frequencies used in the task were relatively closely spaced, which may have reduced sensitivity to frequency-specific (tonotopic) organization of the auditory cortex at the spatial resolution achievable with fMRI.

In addition, caregiver education differed between CPHIV and the control group at a trend level. Because caregiver education may influence children's cognitive development (Do and McCoy, 2025), this imbalance represents a potentially confounding factor. Although caregiver education was not included as a covariate in the present analyses, future studies should incorporate this variable to better isolate HIV-related effects on auditory processing.

Finally, the present study cannot disentangle the relative contributions of HIV infection, prolonged ART exposure, early-life nutritional status, and infectious burden to the observed differences in auditory cortex activation. Addressing these interrelated factors will require longitudinal studies that integrate neuroimaging with detailed clinical, nutritional, inflammatory, and treatment-related measures.

### 4.5 Conclusion

In summary, pure-tone stimulation elicited robust activation of canonical auditory cortices in all children combined. However, CPHIV exhibited reduced bilateral auditory cortex responses relative to children without HIV. These findings suggest that alterations in central auditory processing may contribute to auditory vulnerability in CPHIV, even in the absence of clinically defined hearing loss.

## Data Availability

The raw data supporting the conclusions of this article will be made available by the authors, without undue reservation.
